# Hepatitis B virus infections and risk factors among the general population in Anhui Province, China: an epidemiological study

**DOI:** 10.1186/1471-2458-12-272

**Published:** 2012-04-05

**Authors:** Xiaoqing Li, Yingjun Zheng, Adrian Liau, Biao Cai, Dongqing Ye, Feng Huang, Xiaorong Sheng, Fuyang Ge, Liu Xuan, Shun Li, Jing Li

**Affiliations:** 1 Anhui Academy of Medical Sciences, No. 1 Yonghong Road, Luyang District, Hefei, Anhui 230061, People's Republic of China; 2 Indiana University School of Medicine, 410 W. 10th Street, HS 100, Indianapolis, IN 46202, USA; 3 School of Public Health, Anhui Medical University, No. 69 Meishan Road, Shushan District, Hefei, Anhui 230032, People's Republic of China

## Abstract

**Background:**

Hepatitis B is one of the most common infectious diseases in China. The aim of this study was to determine the prevalence of hepatitis B surface antigen (HBsAg) among the general population and the risk factors associated with HBV infection in Anhui province, China.

**Methods:**

A provincial serosurvey was conducted in four cities, and selected through stratified clustering sampling. Data on demographics, immunization history, medical history, family medical history, and life history were collected, along with serum tested for HBsAg. Completed surveys were analysed from 8,875 participants.

**Results:**

Overall prevalence of HBsAg was 7.44%. Using multivariate analysis, older age was a risk factor for HBsAg infection among children younger than 15 years. Among adults 15-59 years old, the risk factors were male gender, a history of surgical operations, at least one HBsAg-positive family member, and non-vaccination. For adults older than 59 years, the risk factor was a blood transfusion history.

**Conclusions:**

Though Anhui province has already reached the national goal of reducing HBsAg prevalence to less than 1% among children younger than 5 years, there are still several risk factors for HBsAg infection among the older population. Immunization programs should continue to focus on adults, and interventions should be taken to reduce risk factors associated with being infected with Hepatitis B.

## Background

In spite of a vaccine available in 1982, the hepatitis B virus (HBV) remains a serious global public health problem. Worldwide, two billion people are currently infected with HBV, 360 million have chronic infections related to HBV, and 600,000 die each year from HBV-related liver disease or hepatocellular carcinoma [1]. Hepatitis B is particularly endemic in China; the 1992 national serosurvey showed a population's prevalence of 9.75% [2]. That same year, 120 million Chinese were HBsAg carriers, 20 million suffered from chronic hepatitis B, and almost 300,000 died annually from HBV-related infections [2,3]. Hepatitis B is a major cause of death for both liver cancer and cirrhosis, two infections with high mortality rates in China [4].

1992 was also the year that the Ministry of Health recommended a nationwide hepatitis B routine immunization [5]. Between 1992 and 2005, there were three successive policies encouraging more parents to have their infants vaccinated [6]. The first policy started in 1992 where parents paid for the vaccine and a user fee. The second policy began in 2002 where parents only paid for the user fee while the vaccine was still freely available. The final policy was introduced in June 2005, where the vaccine was free and the user fee was waived to all parents. By then, China had fully integrated hepatitis B vaccination as part of its routine immunization for infants. As such, by 2006, the prevalence of HBsAg had dropped to 7.2% for those aged 1-59 years [7,8].

Most HBV infections in developed countries result from sexual activity, injecting drug use, or occupational exposure. In developing countries, other causes of infections can include household contact, vertical transmission hemodialysis, transmission from a surgeon [9], and the receipt of organs or blood products [10]. Given these various ways for HBV infection to occur within any general population, control of hepatitis B is one of the highest priorities in China. Thus far, however, no studies have determined the risk factors of HBV infection on a regional level. Knowledge of such findings can equip local governments with ways to modify national immunization policies if needed.

Compared to other provinces, Anhui province, located in east central China, has a medial incidence of HBV infection [11]. To evaluate whether the impact of the hepatitis B vaccination program since 1992 in Anhui province is similar to the rest of the country, this paper examined an updated Hepatitis B serosurvey to (1) measure the prevalence of hepatitis B among the general population and (2) determine the ongoing risk factors for hepatitis B infection.

## Methods

### Study participants and data collection

This cross-sectional study was performed during 2006, and stratified cluster sampling was used to recruit participants from four cities based on the geographic characteristics of Anhui province. Anhui can roughly be divided into three regions: the northern Huaihe river area, the area between Yangtze and Huaihe Rivers, and the southern Yangtze River area. Cluster sampling was based on these three regions. The first level of stratification sampling involved cities: one selected city in the northern Huaihe River area, two in the area between the Yangtze and Huaihe Rivers (a second city was selected as this area was the most populous of the three), and one in the southern Yangtze River area. In these four cities, all streets in the urban areas and all towns in the rural areas were ranked according to three grades (high, medium, and low) based on the socio-economics of the people living in each area. One urban street and one rural town were then selected randomly from each grade; this was the second level of stratification. The final level of stratification was the random selection of communities and administrative villages, with one community for each urban street and one administrative village for each rural town. In total, 8,895 participants were recruited from 12 communities and 12 administrative villages. Participants were eligible as long as their current residence was in one of the selected areas for the survey.

To raise awareness of the HBV serosurvey, all selected residents were informed twice, first by local leaders and second by local doctors, a week before the actual administration. The serosurvey collection for each community or administrative village covered four days. After informed consent was obtained, all participants were interviewed face-to-face by trained staff in a private room. A standard questionnaire was used to compile each participant's demographic information and risk factors.

### Specimen collection and laboratory testing

Blood samples collected from each participant included 5 mL for those aged over 6 years, and 2 mL for children aged 6 years or less. All blood samples were stored at 0°C and transported to the Hepatitis Laboratory of the Department for Bacteria and Viruses at the Anhui Academy of Medical Science in Hefei after each day's surveying. These serum specimens were screened for HBsAg by enzyme-linked immunoabsorbent assays (ELISA). ELISA reagents were purchased from the manufacturer Shanghai Kehua company in China. For specimens with inconsistent results, Abbott EIA reagents were used for reconfirmation testing.

### Statistical analysis

All data were inputted twice into an EPI Data 3.02 software database [12]. After verifying its accuracy, SPSS 10.0 software located at the Epidemiology and Statistics Department of the School of Public Health at Anhui Medical University was used for analysis. The prevalence rates of HBV seromarkers included point estimates. To identify factors that affected the prevalence of HBsAg for all participants, separate multivariate logistic regressions were analysed for populations aged 0-14 years, 15-59 years, and 60+ years. The dependent variable was the prevalence for HBsAg; the independent variables included gender, location of community (urban vs. rural), marriage status, occupation, education, unsafe injections, history of operations, blood transfusion history, family members being HBsAg-positive, immunization history, personal medical history, family medical history, and life history. The significance level was set at *P *< .05.

### Quality control

Specialists from the Anhui provincial Centers for Disease Control (CDC), Anhui Medical University, the First Affiliated Hospital of Anhui Medical University, Anhui provincial Hospital, and the Anhui Academy of Medical Science convened several times to discuss the statistical design, epidemiological investigation, laboratory testing, training, and analysis of this study. A field pilot study was conducted before the actual survey, based on the field survey's Standard Operating Procedures (SOP), and all staff was trained to conduct interviews; collect, store, transport blood specimens; perform testing; and input and analyse data.

### Ethical issues

The survey was approved by the Anhui Academy of Medical Science Ethics Committee. Each participant was informed of the survey's purpose and their right to have their information kept confidential. In addition, interviewing, laboratory testing, and notification of results were provided free to all participants. Test results were enveloped, sealed, and delivered personally to each participant by the local doctor. All participants received small gifts as compensation for their participation.

## Results

### Characteristics of study participants

Almost all the enrolled participants (8,890 of 8,895) completed the questionnaires and 8,882 blood samples were collected. Completed questionnaires and blood samples resulted in 8,875 eligible datasets (99.8%). The overall prevalence of HBsAg was 7.44%.

Characteristics of the participants are shown in Table 1. Nearly three-quarters (72.9%) were aged 15-59 years. The male to female ratio was 0.76:1, and 99.7% were of Han ethnicity. The percentages of participants who reported having received hepatitis B vaccination were higher among children less than 15 years old (82.9%) than adults aged 15-59 years (29.9%) and those over 60 years of age (5.8%).

**Table 1:** Characteristics of study participants

Variable		Frequency	Percentage
Age (years)	0-4	318	3.6
	5-14	656	7.4
	15-59	6 472	72.9
	60+	1 429	16.1
Urban/Rural	Urban	4 285	48.3
	Rural	4 590	51.7
Gender	Male	3 838	43.2
	Female	5 037	56.8
Ethnicity	Han	8 845	99.7
	Other	30	0.3
Occupation	Worker	2 332	29.5
	Farmer	3 208	40.6
	Student	1 155	14.6
	Civil servant	516	6.5
	Health worker	20	0.3
	Service worker	201	2.5
	Other	469	5.9
Education	Illiterate	2 119	26.8
	Primary school	1 318	16.7
	Middle school	2 078	26.3
	High school	1 304	16.5
	College	1 082	13.7
Marriage status	Unmarried	1 186	15.0
	Married	6 173	78.1
	Dissociation/Loss of spouse	542	6.9
Vaccinated (0-14)	Yes	807	82.9
	No	167	17.1
Vaccinated (15-59)	Yes	1 934	29.9
Variable		Frequency	Percentage
	No	4 538	70.1
Vaccinated (60+)	Yes	83	5.8
	No	1 346	94.2

### Prevalence of HBsAg by demographic characteristics

**Figure 1:**
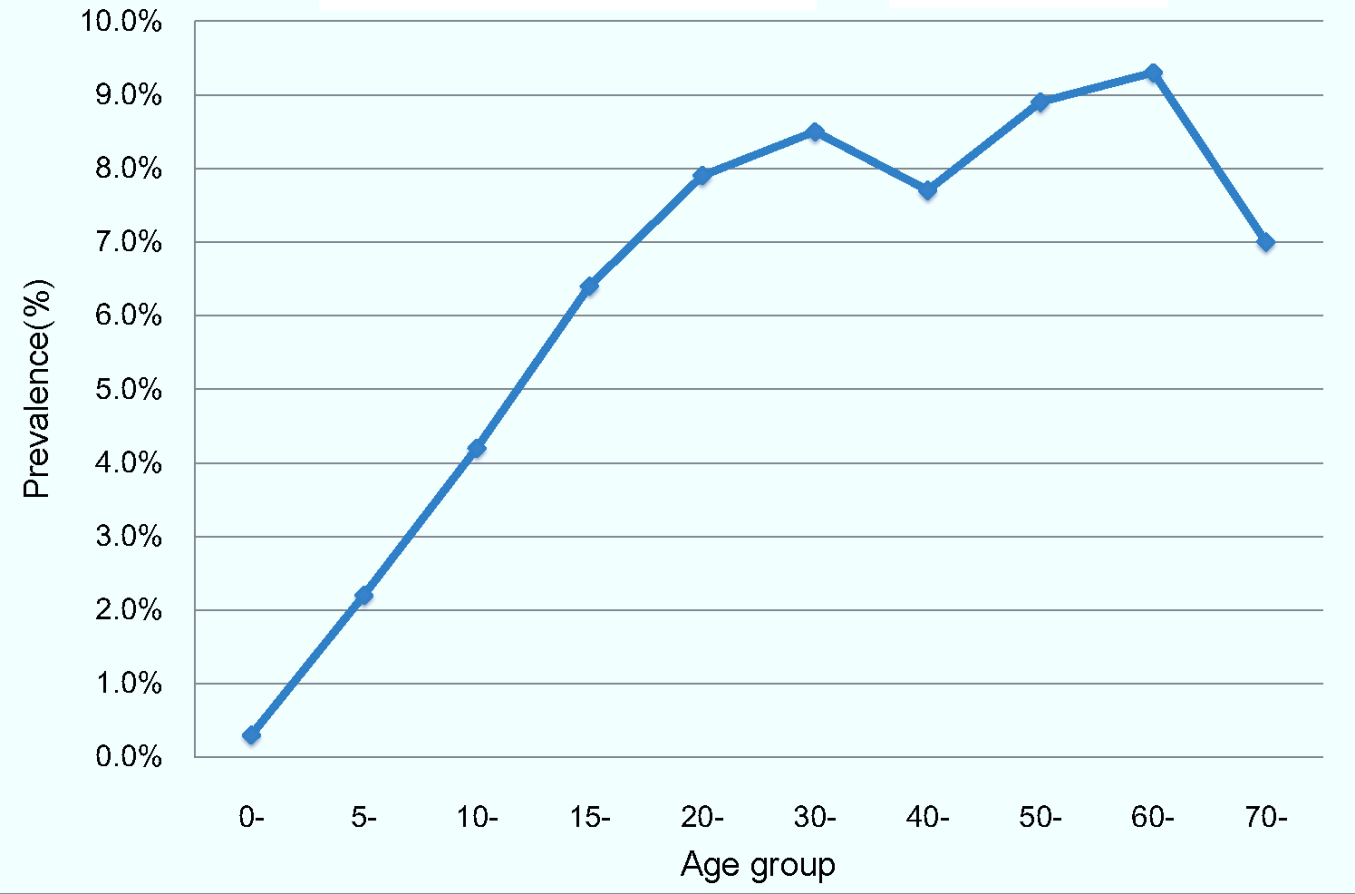
**HBsAg prevalence among participants by age group**.

Figure 1 shows that the prevalence of HBsAg varied by age group. Prevalence was lowest among children less than 5 years old (0.3%) and highest among those aged 60-69 years (9.3%).

The prevalence of HBsAg was significantly higher for males (8.2%) than for females (6.8%, *P *< 0.01) (Table 2). The only other significant finding was that the prevalence of HBsAg among vaccinated participants was lower (6.2%) than among unvaccinated participants (8.7%, *P *< 0.01).

**Table 2:** HBsAg prevalence by demographic characteristics

Variable		Frequency	Prevalence (%)
Urban/Rural	Urban	4 285	7.5
	Rural	4 590	7.4
Gender	Male	3 838	8.2*
	Female	5 037	6.8
Ethnicity	Han	8 845	7.4
	Other	30	10.0
Occupation (for age group 15-59 years)	Worker	2 331	8.7
	Farmer	2 083	8.2
	Student	1 155	6.1
	Civil servant	428	7.5
	Health worker	14	14.3
	Service worker	177	5.6
	Other	284	10.2
Occupation (60+)	Worker	1	0.0
	Farmer	1 125	8.6
	Civil servant	88	4.5
	Health worker	6	16.7
	Service worker	24	0.0
	Other	185	9.7
Education (aged 15-59 years)	Illiterate	1 304	7.1
	Primary school	953	8.7
	Middle school	1 908	8.2
	Middle school		
	High school	1 247	9.1
	College	1 060	6.7
Education (60+)	Illiterate	815	7.6
	Primary school	365	9.9
	Middle school	170	8.2
	High school	57	10.5
	College	22	9.1
Marriage status (15-59)	Unmarried	1 161	7.4
	Married	5 137	7.9
	Widowed	174	13.2
Marriage status (60+)	Unmarried	25	8.0
	Married	1 036	8.8
	Widowed	368	7.3
Vaccinated (0-14)	Yes	807	2.0
	No	167	4.2
Vaccinated (15+)	Yes	2 017	6.2*
	No	5 884	8.7

* *P *< 0.01

### Multivariate analyses of HBsAg status

Among the 0-14 age group, the only significant predictor was age: children aged 10-14 years had a significantly higher prevalence of HBsAg than children aged 5-9 years (OR = 13.78, *P *< 0.05), and children aged 5-9 years had a higher prevalence for HBsAg than children aged 0-4 years (OR = 7.15, *P *< 0.10) (Table 3).

**Table 3:** Multivariate logistic regression analysis of HBsAg prevalence for participants aged 0-14 years

Variable	Category	OR	95% CI for OR		*P *value
				
			Lower	Upper	
Age group	0-4*	1			
	5-9	7.15	0.86	59.77	0.069
	10-14	13.78	1.82	104.51	0.011

*Reference category; Adjusted variables: gender, location of community, immunization history.

For participants aged 15-59 years, several factors significantly contributed to having HBsAg: gender (male), (OR = 1.47, *P *< 0.01), a history of surgical operations (OR = 1.24, *P *< 0.05), having at least one HBsAg-positive family member (OR = 2.04, *P *< 0.01), and being unvaccinated (OR = 1.51, *P *< 0.01) (Table 4).

**Table 4:** Multivariate logistic regression analysis of HBsAg prevalence for participants aged 15-59 years

Variable	Category	OR	95% CI for OR		*P *value
				
			Lower	Upper	
Gender	Male*	1			
	Female	0.68	0.57	0.82	< 0.001
Unsafe injections	No*	1			
	Yes	1.23	0.99	1.51	0.053
History of operations	No *	1			
	Yes	1.24	1.00	1.53	0.048
Blood transfusion history	No*	1			
	Yes	1.57	0.98	2.53	0.060
Family member is HBsAg positive	No*	1			
	Yes	2.04	1.55	2.68	< 0.001
Immunization history	No*	1			
	Yes	0.66	0.53	0.83	< 0.001

*Reference category; Adjusted variables: location of community, marriage status, occupation, education, family medical history, and life history.

Among adults aged 60+ years, the only significant predictor of HBsAg was having any blood transfusion history (OR = 2.48, *P *< 0.05) (Table 5).

**Table 5:** Multivariate logistic regression analysis of HBsAg prevalence for participants aged 60+ years

Variable	Category	OR	95% CI for OR		*P *value
				
			Lower	Upper	
Unsafe injections	No*	1			
	Yes	1.76	0.97	3.18	0.06
Blood transfusion history	No*	1			
	Yes	2.48	1.07	5.76	0.04

*Reference category; Adjusted variables: gender, location of community, marriage status, occupation, education, history of operations, family members being HBsAg-positive, immunization history, personal medical history, family medical history, and life history.

## Discussion

In 1992, the World Health Organization (WHO) recommended the integration of the hepatitis B vaccine into the national immunization programs of all highly endemic countries by 1995 [13]. Following the WHO's recommendation, the HBsAg prevalence in China fell among children born after 1992 [7] and more so for children born after 2002, when the hepatitis B vaccine was fully integrated into routine infant immunization [7]. Our study confirms those findings. In Anhui province, HBsAg prevalence among children aged 0-4 years is merely 0.3%, which is lower than children aged 5 and up.

The carrier rate for HBsAg is 7.44% among all participants. To compare this figure with the national serosurvey, the carrier rate in our paper was standardized based on the 2002 national population constituent ratio, which was 6.56%. The standardized rate for HBsAg prevalence among the general population aged 1-59 years is 6.71%, which is lower than the national serosurvey rate in 2006 and lower than the rates from the Anhui serosurveys in 1979 and 1992. Again, we attribute the falling rates to the universal immunization program for infants. The prevalence of HBsAg is low among children age < 5 years but this increases with age, which indicates an inverse relationship between age and likelihood of vaccination. This suggests the need for an immunization program to encourage both older children and adult populations to be vaccinated as well. The Chinese government is currently implementing policies aimed at these populations to control hepatitis B, such as HBsAg screening of blood for transfusion, control of blood exposure in medical settings, and the management and treatment of HBV-infected persons [14].

The multivariate logistic regression analysis revealed that among children, the main risk factor for HBsAg positivity is age. Children aged 0-4 have more access to the hepatitis B vaccine and a better hepatitis B vaccine service than children aged 5 onwards. Thus is because the routine Expanded Programme on Immunization (EPI) has both improved and been more emphasized since 2002. In addition, the financial barriers to vaccination have become less of an issue because of the recent rising economic development in Anhui province. More pregnant women are being screened for HBsAg, and better medical records are being kept. This allows a hospital to determine if the expectant mother requires testing if HBsAg screening was not previously performed. The infants of those women found positive will then be given the HBsAg vaccine and Hepatitis B Immunoglobulin (HBIG) within 24 hours after birth.

Among adults aged 16-59 years, the risk factors include being male, a history of operations, a family member who is HBsAg positive, and not being immunized. These factors are in agreement with recent seroprevalence studies [15,16]. Among adults aged 60+ years old, the greatest risk factor was a history of blood transfusions. This finding suggests that horizontal HBV transmission and health-care-related factors are mainly responsible for the prevalence among all adults. Horizontal transmission and mother-to-infant transmission of HBV are demonstrated by strong family clustering occurring through frequent exposure to blood (e.g., through contact with skin lesions), saliva (e.g., through sharing of toothbrushes and candy), or breast milk [17,18]. These factors are not specific to resource-poor settings, as it has also been described within families living in the United States and Europe [19-21].

Health-care-related transmission has long been recognized as an important source of new HBV infections worldwide. While most HBV transmissions have occurred during invasive surgical or obstetric procedures [1], our findings indicate that people with any operational history are also at risk for HBV infection. There are three possible routes for infection during an operation: the first from a surgeon to a patient, the second from contaminated surgical instruments to a patient, and the third from an HBV-positive patient to another patient staying in the same hospital room [22-24]. Throughout China, nosocomial infection control is conducted stringently in provincial and municipal level hospitals but insufficiently in certain county and town level hospitals as well as several private hospitals where invasive surgical or obstetric procedures are frequently performed [25,26]. Surgeon-to-patient transmissions of hepatitis B were essentially eliminated when the vaccination of health care workers became routine [27]. However, preventing all health-care-related transmissions of HBV requires a comprehensive approach that includes administering nosocomial infections, consistently providing hepatitis B vaccination to healthcare personnel, enforcing stricter measures to reduce blood exposure between healthcare workers and patients, and having all surgical teams committed to promoting and maintaining a safe work environment constantly [28,29].

An interesting finding is that a history of blood transfusions is the greatest risk factor for HBV infection among adults who are more than 59 years old. Although recent investigations have suggested that blood transfusion remains a major risk factor, at least partly due to the presence of occult HBV infection (OBI) among blood donors [30], blood transfusions have become safer in recent decades in China due to the issuing of laws and regulations [31]. The Ministry of Health initially introduced routine strict serum HBsAg screening in all blood centres in the early 1980s. By 1998, the *Law for Donating Blood *was issued, enacting regulations for the *Management of Blood in Clinical Facility *and further enhancing the safety of blood transfusions and safety in the use of serum related products. Transfusion-related HBV infection caused by unsafe blood has been satisfactorily controlled thereafter [31]. However, serological screening of blood donors was first performed in a few blood centres as early as in the late 1970s, and many people could have been infected then before regulations were established. As such, the higher prevalence rates among the elderly could in part be attributed to previous contaminated blood transfusions.

The carrier rate for HBsAg was 2.0% among vaccinated participants aged 0-14 years, and 6.2% over 15 years. This suggests that 141 vaccinated participants failed to respond to the hepatitis B vaccine, and became sero-positive for HBsAg. One possibility is that hepatitis B vaccine did not have an effect on certain people. Another is that several hepatitis B vaccines were not preserved well in cool chains, causing it to be ineffective. A third may be that a number of HBV infected persons did not know their infected status. Although the number is small, failed responses to the vaccine may warrant further investigation.

One limitation of our study is that HBsAg prevalence may be overestimated among children and underestimated among adults because younger participants with HBV infection were more likely to be brought to the survey site by their parents or other guardians, while vaccinated adult participants were less likely to take part in our survey. Another is recall bias. Participants were first asked, "Were you vaccinated with the hepatitis B vaccine?", followed by, "Did you receive 3 injections?", and finally, "When were you vaccinated?" If the answers for the first two questions are both "yes," the participant will be included in the vaccinated variable. Thus, some unvaccinated participants may end up becoming vaccinated participants.

## Conclusions

The integration of the hepatitis B vaccine into China's routine immunization programs has significantly decreased the HBsAg carrier rate among children born after 1992 in Anhui province. In order to reduce HBV infections further, the routine immunization programs among children need to be continued and mass immunization should be emphasized for all. These steps would reduce the circulation of the virus in the community and allow the average age at infection among those who were not immunized to increase. In addition, health education should focus more on dealing with HBV-positive family members, and nosocomial infections and blood transfusion safety should be strictly controlled and ensured by all hospitals and the local governments. Careful screening of blood products for hepatitis B should continue to be emphasized, and new strategies for HBV blood donor screening be applied universally [32]. The management of unresponsive subjects should be strengthened [33], and the need for routine booster doses should be improved among adults for future vaccination policies.

## Competing interests

The authors declare that they have no competing interests.

## Authors' contributions

XL contributed to the study design, was in charge of the field research and data analysis, and drafted the manuscript.

YZ conducted the field research, data analysis and drafted the manuscript.

AL drafted the manuscript.

BC conducted the field research and drafted the manuscript.

DY conducted the field research and drafted the manuscript.

FH conducted the field research and the data analysis.

XS conducted the field research and the laboratory work.

FG conducted the field research and the data analysis.

LX conducted the laboratory work.

SL conducted the field research.

JL conducted the field research.

## Pre-publication history

The pre-publication history for this paper can be accessed here:

http://www.biomedcentral.com/1471-2458/12/272/prepub
